# Consumer Attitude towards Genetically Modified Foods in Iran: Application of Three-Dimensional Model of Corporate Social Responsibility

**DOI:** 10.3390/foods12071553

**Published:** 2023-04-06

**Authors:** Morteza Akbari, Zahra Fozouni Ardekani, Giovanni Pino, Naser Valizadeh, Mostafa Karbasioun, Hamid Padash

**Affiliations:** 1 Faculty of Entrepreneurship, University of Tehran, Tehran 1439813141, Iran; padash@ut.ac.ir; 2 Department of Agricultural Extension and Education, Faculty of Agriculture, Tarbiat Modares University, Tehran 1439813141, Iran; zfozuni@yahoo.com; 3 Department of Management and Economics, University of Salento, 73100 Lecce, Italy; giovanni.pino@unisalento.it; 4 Department of Agricultural Extension and Education, School of Agriculture, Shiraz University, Shiraz 7144165186, Iran; n.valizadeh@shirazu.ac.ir; 5 Faculty of Agriculture, University of Shahrekord, Shahrekord 64165478, Iran; mostafa.karbasioun@gmail.com

**Keywords:** CSR, environmental concern, GM foods, health concern, trust

## Abstract

Although GM food production is considered an important strategy to meet the growing food needs of the population around the world, a majority of the GM food consumers express doubts about purchasing and eating them. However, it can be argued that consumers have different opinions about GM foods and their influence on human health and the natural environment. GM food producer Corporate Social Responsibility (CSR) may significantly affect such opinions, but the effect of this variable has been partially neglected in previous research studies. To address this gap, the present study investigates Iranian consumers’ concerns about GM foods, trust in these products, and perception of GM food producer CSR as determinants of attitudes towards GM food. Data were collected from Iranian consumers. A cross-sectional survey research with a multi-stage random sampling approach was employed to capture the responses of 372 Iranian consumers. The results showed that consumers have both negative and positive attitudes towards GM foods. Perceived social equity, trust, and health concerns were the most important determinants of attitude towards GM foods. According to the results, these variables could account for 52.9% (Cox and Snell R^2^) and up to 70.6% (Nagelkerke R^2^) of the variance of the dependent variable. Furthermore, results revealed statistically significant differences among the consumers with different educational levels in terms of perceived social equity, perceived environmental responsibility, and environmental concern. The research contributes to the body of knowledge in GM food consumption by evolving the CSR to assess attitudes of users concerning GM foods.

## 1. Introduction

Today, food security and the health of people are some of the main challenges all over the world [1,2,3]. Although GM agriculture is considered as key strategy to increase the quantity of food production [4,5,6], GM foods are still a subject of debate among scientists and policy-makers around the world [7,8,9]. On the one hand, the increasing need for sustainable production and consumption [10] has fostered producer and government interest in the application of genetic engineering to agricultural products [11,12]. Many sources have voiced concerns around the possible adverse impacts of consumption and production of GM foods on the environment, food security, public health, and sustainability. These concerns have negatively influenced consumer perception of such products [13,14,15,16]. As a result, consumers have developed some negative attitudes towards GM foods [8,9,17,18]. Consumer concerns are mainly rooted in lack of trust in production process of such products as well as the organizations involved in GM food production [19]. Therefore, it can be concluded that GM food producer CSR could play an important role in reducing such concerns [20]. However, research background on the impact of CSR on attitudes towards GM food is extremely limited [21,22,23]. Previous research studies have proved that CSR have a significant impact on user attitudes [24,25]. Consistently, some studies [24] suggest that consumer perceptions of GM food producer CSR may significantly affect their attitudes towards GM food. However, due to the limited number of investigations [24,25,26] in this field, it is still unclear which specific dimensions of CSR may mostly affect consumer attitudes towards GM foods. 

Previous research studies have employed the well-established Theory of Planned Behavior [23,27,28] to study consumer attitudes towards such products. However, these studies rarely focused on consumer perception about GM food producer CSR [21,22,24,29]. More specifically, previous studies have never applied a three-dimensional model of CSR, which relies on social, economic, and environmental dimensions of responsibility to GM food consumption [14,20,22,30,31,32,33]. Furthermore, very few studies have focused on developing countries such as Iran, where the consumers have scarce information about GM foods [29,34,35,36,37,38,39,40,41]. Due to the significant role of consumers in the adoption and diffusion of GM foods, this study examined Iranian consumers’ attitudes towards GM foods, which mainly depend on their concerns and trust in the GM food industry as well as their attitude about GM food producer CSR. 

In other words, to contribute to the growing literature on GM food, the present research investigates whether and how consumer concerns around GM foods, trust in the GM food industry, and perception about producer CSR affect their attitudes towards such products. To achieve this goal, a quantitative survey method was employed to capture the required data from 372 consumers in Tehran (Iran). GM breeds were first introduced in Iran to improve crop productivity and resistance. However, they have always been an object of disagreements between policy-makers, scientists, and the public community [39,40,41,42,43,44]. Some scholars and thinkers believe that acceptance of international agreements on biosafety such as the Cartagena Protocol could increase public trust in these products. Despite the acceptance of this protocol, it can be claimed that disagreements over this issue have not significantly reduced [45,46].

This study contributes to theory and practice in different ways. Previous literature identified a wide range of concerns related to GM food consumption [46,47,48,49,50,51,52]; however, health and environmental responsibility are some of the most important concerns related to food security [53,54] which the present study attempts to focus on. Moreover, this study also considers consumer trust in GM food producers and other organizations involved in GM food production (such as researchers/specialists, food organizations/institutions, and the government). The effect of this variable on consumer attitude has rarely been investigated in previous studies. Trust in the abovementioned institutions and organizations may positively affect consumer attitudes and intentions towards GM foods and perceptions of their associated risks and advantages [11,14,31,46,55].In addition, past studies documented a positive impact of CSR on consumer attitudes and intentions [21,22,23,56,57]. However, the three-dimensional model of CSR (social equity and environmental responsibility) has rarely been applied by the researchers, despite the fact that it provides a more accurate and realistic picture of consumer perception of producer CSR. In the particular context of GM food consumption, Pino et al. used Carroll’s model and discovered that legal responsibility shapes consumer intention towards GM food, whereas philanthropic responsibility shapes consumer attitudes towards such products [24]. Finally, some other models of CSR such as sustainability-based three-dimensional model of Alvarado-Herrera et al. have recently been developed, which has never been applied to GM food consumption to the best of our knowledge [30]. Building on these studies, present research aimed to investigate the impact of consumer perception of GM food producer CSR on consumer attitudes towards GM food by considering the sustainability-based model of CSR. 

## 2. Materials and Methods

**Figure 1:**
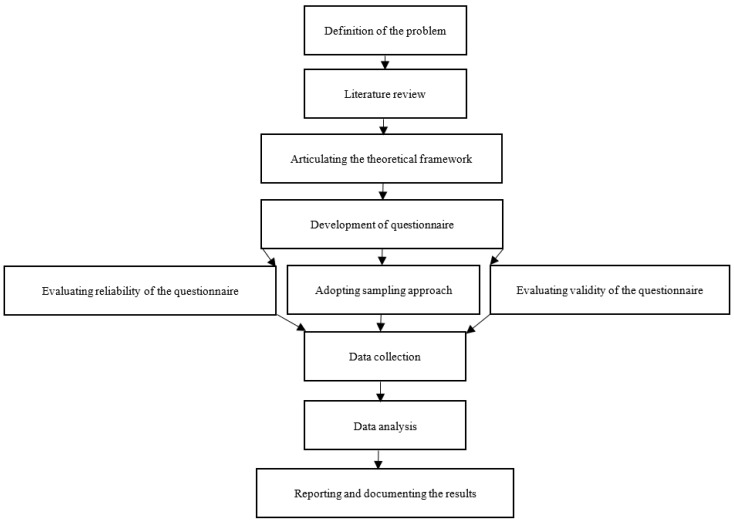
Research procedure and process.

In general, Figure 1 summarizes the main steps and procedure of present study. However, in order to clarify the research methods, we explain the population and sampling, research instrument and definitions of constructs, reliability and validity, classification method of the dependent variable, and data analysis and demographics of the respondents in the following section. 

### 2.1. Population and Sampling

The required data were collected from Tehran, Iran, an administrative region with over 15 million inhabitants that includes 22 urban districts. Through a multi-stage random sampling approach, 550 questionnaires were sent out to the cases, who were residents of these districts (6 out of 22 districts). The data were collected using a questionnaire. As the selected people were likely to be informed about GM food, they were selected to constitute the population of this research. We focused on such consumers because other community members were likely to follow this consumer group’s attitude towards GM products. Ultimately, 372 completed questionnaires were collected. 

### 2.2. Research Instrument and Definitions of Constructs

The research questionnaire was developed based on our literature review and feedback from experts in the research area. This questionnaire assessed the constructs on a five-point Likert scale (1 = Strongly disagree; 5 = Strongly agree). The questionnaire included a 14-item scale to assess respondent perceptions of GM food producer CSR (perceived economic responsibility, perceived social equity, and perceived environmental responsibility). More specifically, a four-item scale was adapted from Pino et al. [24] to measure perceived economic responsibility. A five-item scale was derived from Alvarado-Herrera to assess perceived social equity [30]. Similarly, a five-item scale was derived from Alvarado-Herrera et al., which was employed to assess perceived environmental responsibility [30]. Health concern construct was measured using a six-item scale adapted from Kikulwe et al. [14] and Montuori et al. [58]. In addition, environmental concern was measured through a six-item scale adapted from [29]. Trust in the GM industry was measured with a three-item scale adapted from [14,15,16,17,18,19,20,21,22,23,24,25,26,27,28,29,30,31,32,33,34,35,36,37,38,39,40,41,42,43,44,45,46,47,48,49,50,51,52,53,54,55]. Finally, attitude towards GM food was measured using a six-item scale adapted from previous studies [55,56,57,58,59]. The items employed for each construct are presented in Table 1.

### 2.3. Reliability and Validity

The validity of the indices was established by a group of academic experts in the fields of food sciences, medical sciences, and social sciences. Measures of internal consistency were satisfactory. Accordingly, Cronbach’s alpha values for the constructs were as follows: perceived economic responsibility (four items) = 0.83, perceived social equity (five items) = 0.91, perceived environmental responsibility (five items) = 0.92, health concern (six items) = 0.78, environmental concern (six items) = 0.90, trust (five items) = 0.63, and attitude (eight items) = 0.93. 

### 2.4. Classification Method of the Dependent Variable

Respondent attitude towards GM foods was used to identify two groups: a “positive attitude” group (coded with 1), whose attitude was more positive than the overall sample’s average attitude; and a “negative attitude” group (coded with 0), whose attitude was less positive than the overall sample’s average attitude. 

### 2.5. Data Analysis and Demographics of the Respondents

Table 2 illustrates the respondents’ demographic features. In sum, means, standard deviation, *t*-test, one-way ANOVA, and binary logistic regression were applied in this stage.

## 3. Results

The mean values of GM food producer economic responsibility (M = 3.58), respondent health concern (M = 3.62), and environmental concern (M = 4.36) were higher than the scale mid-point (Table 3). However, the mean values for the perceptions of GM food producer social equity (M = 2.74), perceived environmental responsibility (M = 2.84), and trust in the GM food industry (M = 3.22) were lower than the scale mid-point. In the negative attitude group, the mean values of perceived economic responsibility (M = 3.44), health concern (M = 4.04), and environmental concern (M = 4.43) were considerably high. Similarly, in the positive attitude group, the mean values of all variables except for health concerns were at high levels. These findings denote that both groups believe in financial profits as a focal objective of GM food producers and related organizations. Such organizations pay little attention to concerns around the influence of GM foods on consumer health and the environment. Therefore, from the perspective of the respondents, consuming these products may harm human health and the environment.

Concerns about GM foods are quite perceivable in the respondents’ point of views, even in those with positive attitudes. The positive group tends to trust GM food producers and feel that they consider social equity and environmental responsibility in their products. Their mean values of perceived social equity and perceived environmental responsibility were relatively low. These results show that from the perspective of these consumers, paying attention to the social and environmental dimensions of CSR in food-based issues is still limited in Iran. Employing the independent sample *t*-test (two-tailed) revealed an important difference (*p* < 0.01) between two attitudinal groups in terms of perceived economic responsibility, perceived social equity, perceived environmental responsibility, and health concern (Table 3). However, no significant difference was detected among these groups in terms of environmental concern.

### 3.1. Comparative Analysis Based on the Education Level

Educational differences/similarities of respondents were assessed through a one-way ANOVA. Table 4 reveals that consumers were significantly different in perceived environmental responsibility (*p* < 0.01), perceived social equity, and environmental concern (*p* < 0.05). Regarding perceived social equity, the post hoc (LSD) test showed that significant differences existed among B.Sc. (M = 15.20), M.Sc. (M = 13.20), and Ph.D. (M = 12.70) groups. Similar results were achieved for the constructs of perceived environmental responsibility, and statistically significant differences were identified among B.Sc. (M = 15.70), M.Sc. (M = 13.87) and Ph.D. (M = 12.82) groups. Furthermore, significant differences were detected between B.Sc. (M = 25.30) and M.Sc. (M = 26.75) groups in terms of environmental concern. These results suggest that respondents with high educational levels believe that the commitment of GM producers to social equity and environmental responsibility is low. Furthermore, these consumers appear more concerned about the environment than the B.Sc. group (consumers with low education level).

Such results may derive from the fact that consumers with higher educational levels are likely to be more informed about GM technology and GM products. Moreover, the manifestation of greater environmental concerns within the M.Sc. group suggests that these viewpoints about the social commitment of GM producers and the related organizations may provoke a lack of trust in the GM food industry among the more educated consumers.

### 3.2. Binary Logistic Regression (BLR) Analysis

To investigate the effect of the studied constructs on consumer attitudes towards GM foods, six factors were assumed as independent variables of a binary logistic regression analysis. In this analysis, respondent attitude towards GM food was considered as a binary dependent variable. Logistic regression analysis was implemented using a forward stepwise (likelihood ratio) method. Table 5 indicates the result model. The model accounted for 52.9% (Cox and Snell R^2^) and up to 70.6% (Nagelkerke R^2^) of the variance of the dependent variable. Only three of the alleged predictors including perceived social equity (b = 0.222, *p* < 0.05), trust (b = 0.268, *p* < 0.01) and health concern (b = −0.323, *p* < 0.01) were determined to have statistically significant effects on the attitude. Accordingly, perceived social equity and trust showed positive effects on attitude, while health concerns had a negative influence on the attitude of consumers. These findings denote that paying attention to the social equity and increasing trust in the GM food industry could improve consumer attitudes towards GM foods. On the other hand, consumer health concern regarding GM foods reduces the probability of consuming these foods. The accuracy value of the model was high enough to present an appropriate classification in both groups. It was higher in the positive attitude group (88.9%) than in the negative attitude group (88.5%). Nevertheless, the overall classification accuracy of the model was considerable (88.7%).

## 4. Discussion

The aim of this research was to extend the current knowledge of consumer attitudes about GM food. This study contributed to this field of research by developing a framework that has not been tested in previous studies. This framework not only included some established antecedents such as consumer concern for their health and the natural environment and trust in the GM food industry, but also considered consumer perception about the CSR of GM food producers. To this end, we used a three-dimensional model of CSR [30], which had not been applied to analyze the GM food consumer attitudes so far. We determined that the three dimensions of CSR have different levels of importance for Iranian consumers and that social equity, in particular, affects their attitudes towards GM food. Iranian consumers exhibit both negative and positive attitudes towards GM foods; but, consistent with previous studies [8,9,10,24], we discovered that about half of the investigated consumers had a rather negative attitude towards such products. We detected statistically significant differences between the negative and positive consumer groups in terms of their health concern and trust as well as their perception of GM producer social equity.

Findings also revealed that customers with negative attitudes towards GM foods were more concerned about their health and the environment; however, customers with positive attitudes towards GM food were more concerned about the CSR dimensions. It should also be mentioned that this group trusted GM food producers more. The positive attitude group appears concerned about the environmental impact of these foods and producers and related organizations’ excessive attention to the economical aspect of GM food production. Such concerns will probably continue to exist in the future [21,22,24,50]. This study is one of the few of those performed in developing countries that compares consumer groups based on their education level. We detected that there are statistically significant differences in terms of perceived social equity among respondents with B.Sc., M.Sc., and Ph.D. degrees. The B.Sc. group presented higher mean scores. In addition, significant differences were detected among the B.Sc. group with M.Sc. and Ph.D. groups in terms of perceived environmental responsibility. It is worth mentioning that statistically significant differences were detected between B.Sc. and M.Sc. groups with respect to environmental concern. Consumers with higher education perceived less social equity and environmental responsibility; they also exhibited higher levels of environmental concern. This issue can be associated with respondent awareness of GM foods and the consequences of adopting these products. For instance, some studies [50,60,61,62,63] have highlighted the important role that consumer knowledge and educational background play in GM food acceptance.

The results of the BLR showed that although trust in the GM food industry and social equity could positively influence Iranian consumers’ attitudes towards GM foods, health concerns have the opposite effect. Hence, acceptance of GM food could be higher if Iranian consumers would have higher trust in the organizations and the institutions involved in GM food production [11,14,32,63,64,65]. In line with past studies that detected a positive influence of trust on consumer attitudes and perceptions of GM foods [11,14,55,66,67], our results indicated that trust in Iranian government and GM-related organizations could contribute to creation of a favorable attitude towards GM foods. The positive impact of social equity on consumer attitudes demonstrates that Iranian consumers would be more likely to adopt GM foods if such products would benefit not only the industry, but also society. Finally, in line with previous studies that highlighted the negative influence of health-related risks on consumer attitudes towards GM foods, our findings showed that health concerns could represent an important barrier to the diffusion of GM food products.

Our results present several implications for managers and policy-makers. Health concern was high in the negative attitude group and negatively affected the entire sample’s attitude value towards GM food products. Therefore, managers and policy-makers may define food-safety controls and set safety standards and policies on GM food production and importation to ensure that such products are healthy and safe. Specific information campaigns could be run on mass media and provide consumers with clear and easily understandable information about the benefits as well as the known risks related to GM food consumption. Only after taking these steps can consumers decide whether to accept such products or not. The media should deliver general and basic fundamental information about biotechnology to the public to pave the way for presenting objective information about these products and their effects on consumers and the environment. It is expected that this fundamental information could present as the pre-requisite for more specific GM products and prevent the diffusion of uncertain or biased information created by some GM producers. These campaigns could involve informants such as scientists and researchers, since they are often trusted more than other informants [37]. Meanwhile, the government could set appropriate labelling policies and specify the type and amount of information that producers and other organizations in the GM food industry should deliver to consumers.

Finally, our findings also suggest that organizations involved in GM food production should disclose information related to their commitment to the social problems that may be connected with the diffusion of GM food (for instance, disparities in the access to such food or potential improvements in people’s quality of life) to provide consumers with everything they need to form their own opinion about GM foods.

## 5. Conclusions

This study assessed the impact of Iranian consumers’ concerns about GM food, trust in the GM industry, and perceptions of GM food producer CSR on their attitude towards GM foods. To the best of our knowledge, this research is the first study that employs a three-dimensional (economic, social, and environmental responsibility) model of CSR to analyze the consumer attitudes towards GM foods. This study is important from several aspects. First, it helps to identify the antecedents of consumer attitudes towards GM products. Second, it shows with practical suggestions, ways to change or direct the positive and negative attitude towards these products. This is an issue that can be very useful for policy-makers, decision-makers, manufacturers, and behavioral change practitioners. In addition, this study, by measuring the consumer attitudes, trust, and perception towards CSR of producers, can help producers to pay more attention to consumer preferences as an important criterion in their productions. The results demonstrated that perceived economic responsibility, perceived environmental responsibility, and environmental concern did not result in significant predictions. However, perceived social equity and trust in the GM food industry resulted in significantly positive prediction of consumer attitudes towards these products. On the other hand, health concern is the most significant predictor, which negatively affects consumer attitudes towards GM foods. Furthermore, the significant differences were detected among consumers with different education levels in terms of perceived social equity, perceived environmental responsibility, and environmental concern.

This research features some limitations. First, the study sample comprised consumers who mostly lived in urban areas of Iran. Therefore, it is recommended that this research be replicated in un-urban areas. Moreover, future studies are suggested to investigate the influence of other possible factors on attitude towards GM foods to confirm the general validity of the obtained results. Our study used a multi-stage random sampling method among consumers with a high educational level. However, cross-national research could assess whether demographic, geographical, and cultural factors could play a role in the adoption of GM foods. Nowadays, there are impressive investments in GM foods in developing countries. Therefore, future research should investigate the influence of public governmental policies on consumer attitudes and promotion of GM foods.

## Figures and Tables

**Table 1:** Items used for measuring the constructs.

Constructs	Number of Items	Items	Sources
Perceived economic responsibility	4	I believe that GM food producers: 1. maximize profits. 2. control their production costs strictly. 3. plan for their long-term success. 4. always improve economic performance.	[24]
Perceived social equity	5	I believe that GM food producers are really trying to: 1. sponsor public health programs. 2. be highly committed to well-defined ethical principles. 3. sponsor cultural programs. 4. make financial donations to social causes. 5. help to improve quality of life in the local community.	[30]
Perceived environmental responsibility	5	I believe that GM food producers are really trying to: 1. sponsor pro-environmental programs. 2. allocate resources to offer services compatible with the environment. 3. carry out programs to reduce pollution. 4. protect the environment. 5. recycle its waste materials properly.	[30]
Health concerns	4	1. I am concerned about harmful effects of GM food consumption on human health in future. 2. Even though GM food may have advantages, it is basically against nature. 3. GM technology should not be used even for medicinal purposes. 4. GM production can increase the formation of resistant microorganisms. 5. GM production can reduce the number of the vegetable species with a consequent nourishment world damage.	[14,15,16,17,18,19,20,21,22,23,24,25,26,27,28,29,30,31,32,33,34,35,36,37,38,39,40,41,42,43,44,45,46,47,48,49,50,51,52,53,54,55,56,57,58]
Environmental concern	6	1. I am very concerned about the environment. 2. Humans are severely abusing the environment. 3. I would be willing to reduce my consumption to help protect the environment. 4. Major political change is necessary to protect the natural environment. 5. Major social changes are necessary to protect the natural environment. 6. Anti-pollution laws should be enforced more strongly.	[29]
Trust in the GM industry	6	If the majority of people of my country are in favor of GM food, it should be legalized. The government effectively monitors the correct use of GM in the medical, agricultural, and other sectors. I trust in the scientist reports when they show that transgenic food is not harmful. I trust pharmaceutical companies are conscious of their responsibilities in conducting genetic engineering or handling the modified products. I trust agriculture companies are conscious of their responsibilities in conductinggenetic engineering or handling the modified products.	[14,15,16,17,18,19,20,21,22,23,24,25,26,27,28,29,30,31,32,33,34,35,36,37,38,39,40,41,42,43,44,45,46,47,48,49,50,51,52,53,54,55]
Attitude towards GM food	6	I think that purchasing GM food is: 1. interesting. 2. a good idea. 3. important. 4. beneficial 5. wise. 6. favorable.	[55,56,57,58,59]

**Table 2:** Respondents’ demographic characteristics based on their GM attitude.

Variables	Negative Attitude	Positive Attitude
Gender Male Female	% 50.50 49.50	% 50.60 49.40
Income (Rial)	Mean = 4,167,406 SD = 1.85	Mean = 3,337,906 SD = 9.52
Having knowledge concerning GM foods Yes No	% 94.30 5.70	% 77.20 22.80
Family size	Mean = 3.5 SD = 1.57	Mean = 3.88 SD = 2.91
Education B.Sc. M.Sc. Ph.D. Unspecified	% 24.40 47.40 24.00 4.20	% 33.90 42.20 18.90 5.00

**Table 3:** Comparison of consumers with negative and positive attitudes towards GM food.

	Total		Negative Attitude Group		Positive Attitude Group		*t*-Value	*p*
Variables	Mean	SD	Mean	SD	Mean	SD		
Attitude towards GM foods	2.62	1.12	1.9	0.69	3.84	0.49	−31.08	0.00 **
Perceived economic responsibility	3.58	0.73	3.44	0.82	3.74	0.58	−4.07	0.00 **
Perceived social equity	2.74	1.07	2.10	0.94	3.42	0.74	−14.96	0.00 **
Perceived environmental responsibility	2.84	1.14	2.22	1.05	3.49	0.81	−13.08	0.00 **
Health concern	3.62	0.80	4.04	0.66	3.17	0.70	12.27	0.00 **
Environmental concern	4.36	0.72	4.43	0.75	4.30	0.68	1.78	0.076 ^ns^
Trust in the GM food industry	3.22	0.79	2.79	0.65	3.69	0.64	−13.59	0.00 **

Note: ns = Not significant; ** *p* < 0.01.

**Table 4:** Comparison of the variables based on the educational level.

Variables	Mean		F	*p*
Perceived economic responsibility	Between the Groups	0.27	0.032	0.969 ^ns^
	Within the Groups	8.44		
Perceived social equity	Between the Groups	181.21	6.417	0.002 *
	Within the Groups	28.23		
Perceived environmental responsibility	Between the Groups	214.20	6.870	0.001 **
	Within the Groups	31.18		
Trust in the GM food industry	Between the Groups	41.03	2.682	0.070 ^ns^
	Within the Groups	15.30		
Environmental concern	Between the Groups	68.90	3.769	0.024 *
	Within the Groups	18.28		
Health concern	Between the Groups	0.57	0.025	0.976 ^ns^
	Within the Groups	23.25		

* *p* < 0.05; ** *p* < 0.01. ns = not significant.

**Table 5:** Binomial Logistic Regression Analysis for six variables.

Variables	b	S.E.	Wald	*p*	Exp (b)	95% C.I. for EXP (b)	
						Lower	Upper
Perceived economic responsibility	0.112	0.078	2.100	0.147 ^ns^	1.119	0.961	1.303
Perceived social equity	0.222	0.072	9.400	0.002 *	1.248	1.083	1.438
Perceived environmental responsibility	0.063	0.064	0.965	0.326 ^ns^	1.065	0.939	1.208
Trust in the GM food industry	0.268	0.058	21.076	0.00 **	1.308	1.166	1.467
Environmental concern	0.025	0.045	0.294	0.587 ^ns^	1.025	0.938	1.120
Health concern	−0.323	0.052	37.959	0.00 **	0.724	0.654	0.802
Constant	−3.740	1.460	6.559	0.010	0.024	-	

Note: ns = Not significant; * *p* < 0.05; ** *p* < 0.01.

## Data Availability

Data is contained within the article.
